# Mitochondrial Transfer in Cardiovascular Disease: From Mechanisms to Therapeutic Implications

**DOI:** 10.3389/fcvm.2021.771298

**Published:** 2021-11-26

**Authors:** Jun Chen, Jinjie Zhong, Lin-lin Wang, Ying-ying Chen

**Affiliations:** ^1^Department of Basic Medicine Sciences, and Department of Obstetrics of the Second Affiliated Hospital, Zhejiang University School of Medicine, Hangzhou, China; ^2^Department of Basic Medicine Sciences, and Department of Orthopaedics of Sir Run Run Shaw Hospital, Zhejiang University School of Medicine, Hangzhou, China

**Keywords:** cardiovascular disease, mitochondria, mitochondrial transfer, mitochondrial transplantation, tunneling nanotubes, extracellular vesicles

## Abstract

Mitochondrial dysfunction has been proven to play a critical role in the pathogenesis of cardiovascular diseases. The phenomenon of intercellular mitochondrial transfer has been discovered in the cardiovascular system. Studies have shown that cell-to-cell mitochondrial transfer plays an essential role in regulating cardiovascular system development and maintaining normal tissue homeostasis under physiological conditions. In pathological conditions, damaged cells transfer dysfunctional mitochondria toward recipient cells to ask for help and take up exogenous functional mitochondria to alleviate injury. In this review, we summarized the mechanism of mitochondrial transfer in the cardiovascular system and outlined the fate and functional role of donor mitochondria. We also discussed the advantage and challenges of mitochondrial transfer strategies, including cell-based mitochondrial transplantation, extracellular vesicle-based mitochondrial transplantation, and naked mitochondrial transplantation, for the treatment of cardiovascular disorders. We hope this review will provide perspectives on mitochondrial-targeted therapeutics in cardiovascular diseases.

## Introduction

Cardiovascular diseases refer to a group of disorders affecting the heart and blood vessels, including coronary artery disease (such as myocardial infarction), arrhythmia, hypertensive heart disease, valvular heart disease, cardiomyopathy, et al. (1, 2). Mitochondria not only serve as power plants in cells but also act as crucial regulators in many biological processes, including reactive oxygen species (ROS) signaling, redox balance, calcium homeostasis, protein quality control, and programmed cell death (3, 4). The abnormal morphology and dysfunction of mitochondria have been proven as the principal mechanisms in the pathogenesis of cardiovascular diseases, such as heart failure, myocardial infarction, atherosclerosis, and hypertension (4–6). So mitochondria-targeted therapy is suggested to be a potential treatment strategy for cardiovascular diseases. In recent years, a large number of pharmaceutical compounds and nutritional supplements that can boost mitochondrial bioenergetics efficiency have been developed. However, clinical trials of these agents for cardiovascular diseases were hardly approved to carry out, even less to evaluate their clinical effectiveness and safety. The main obstacle is because many protein components of mitochondria are the network hubs of multiple biological pathways. If a chemical compound targeting one of these hubs is used, it can not only modify the anticipated biological pathways but also change other unexpected mitochondrial processes (5). Therefore, patients with cardiovascular diseases would fail to achieve the desired outcomes by using these mitochondrial-targeted drugs (5). Given the complexity of the biological function of mitochondria, researchers have begun to consider rescuing the injured cells through mitochondrial transfer, that is, replacing damaged mitochondria with healthy mitochondria from donor cells.

The intercellular mitochondrial transfer was reported for the first time by Spees and colleagues in 2006. They demonstrated that transferring functional mitochondria of bone marrow-derived stem cells to defective parenchymal cells increases the aerobic respiration capacity of recipient mitochondria (7). Nowadays, more and more studies have revealed that cells in the cardiovascular system (such as cardiomyocytes, vascular smooth muscle cells, endothelial cells, et al.) can act as donors or recipients during mitochondrial transfer under physiological conditions (8–12). However, harmful stimuli (such as ischemia-reperfusion, oxidative stress, and toxic chemicals) can change the direction and efficiency of intercellular mitochondrial transfer. Studies have shown that cells can eliminate defective mitochondria by delivering them to recipient cells (such as macrophages) to maintain homeostasis. And the released mitochondria can also act as a distress signal to activate the rescue properties of recipient cells (12, 13). Meanwhile, damaged cells can take up exogenous functional mitochondria and integrate them into endogenous mitochondria networks, which improve their biological process and enhance their repairability (14, 15). In this review, we summarized the mechanism and function of mitochondrial transfer in the cardiovascular system. We also discussed the advantages and challenges of mitochondrial transfer strategies in the treatment of cardiovascular disorders. We hope this review will provide perspectives on mitochondrial-targeted therapeutics in cardiovascular diseases.

## Mechanisms of Intercellular Mitochondrial Transfer

Intercellular transfer of mitochondria in the cardiovascular system is through several pathways, including tunneling nanotubes (TNTs), extracellular vesicles (EVs), naked mitochondria extrusion, and others.

### Mitochondrial Transfer *via* Tunneling Nanotubes

**Figure 1:**
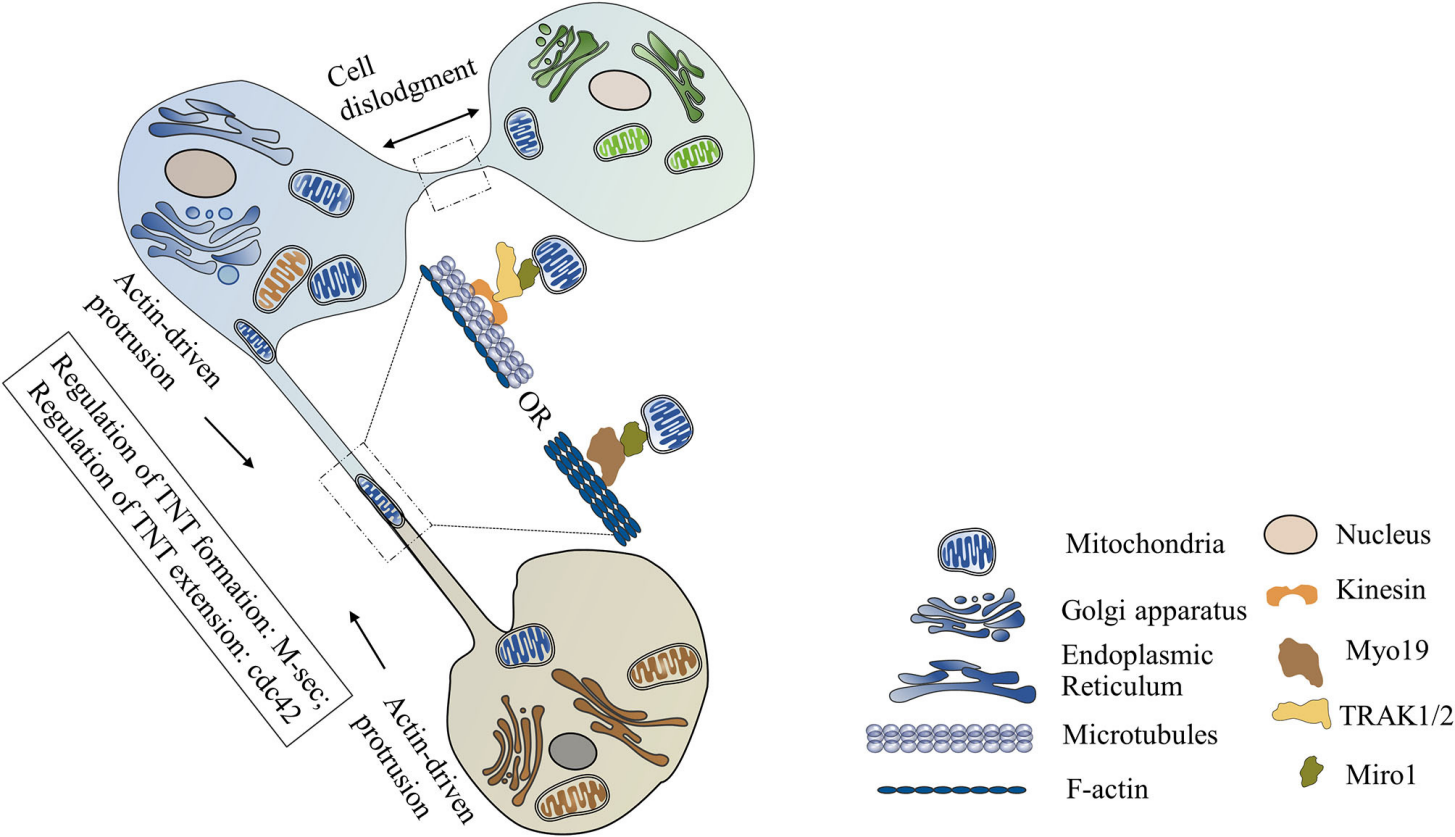
Mitochondrial transfer *via* tunneling nanotubes (TNTs). TNTs are formed *via* cell dislodgment mechanism or actin-driven protrusion mechanism. TNTs containing only actin are small in diameter. TNTs containing both F-actin and microtubules are large in diameter. M-Sec is necessary for the formation of TNTs, while Cdc42 is required for the extension of TNTs. Mitochondrial Rho GTPase 1 (Miro1), a tail-anchored mitochondrial outer membrane protein, plays a critical role in mediating mitochondrial movement along the TNTs. After combing with the adaptor protein TRAK1/2, Miro1 can recruit motor protein kinesin and initiate microtubule-based mitochondrial movement. Miro1 can also mediate actin-based mitochondrial transport *via* binding with motor protein Myo19.

TNTs, also called membrane nanotubes, are long tubular membrane structures (Figure 1). TNTs were discovered as unique structures for intercellular communication for the first time by Rustom and coworkers in 2004 (16). Recent studies have shown that cells in cardiovascular systems (such as cardiomyocytes, cardiac fibroblasts, endothelial cells, and vascular smooth muscle cells) can exchange mitochondria with their neighboring cells *via* TNTs (Table 1) (8–12, 15, 17–24). The intercellular transfer of mitochondria through TNTs could be unidirectional or bidirectional. The diameter of TNTs ranges from 50 to 1,000 nm (8, 17, 21, 22, 24). The length of TNTs, which differs in various types of cells, is usually 5–120 μm (8, 9, 17). Actin is the principal component of TNTs, and filamentous actin (F-actin) polymerization is necessary for the assembly of TNTs (15, 18, 23). Besides actin, another cytoskeleton component, microtubule, is also found in some TNTs (23). Both F-actin and microtubules could act as cytoskeletal tracks for the movement of mitochondria. TNTs containing both microtubules and F-actin are large in diameter (>0.7 μm) and responsible for the long-distance delivery of mitochondria. TNTs containing only actin are small in diameter (<0.7 μm) and in charge of the short-distance transport of mitochondria (19, 21, 25–28).

**Table 1:** Properties of mitochondrial transfer-related TNTs in cardiovascular system.

**Donor cells**	**Recipient cells**	**Cytoskeleton compounds**	**Diameter**	**Length**	**Stimulus**	**References**
**Bidirectional mitochondrial transfer**						
MSCs	Cardiomyocytes or cardiac myoblasts	F-actin	200–500 nm	–	Physiological condition, hypoxia, doxorubicin, tumor necrosis factor-α	(15, 17)
MSCs	Vascular smooth muscle cells	F-actin	–	–	Physiological condition	(10)
MSCs	HUVECs	F-actin, or both F-actin and microtubules	–	–	Bidirectional (physiological condition), unidirectional (hypoxia, cytarabine)	(12, 18)
Cardiomyocytes	Cardiac fibroblasts	F-actin and microtubules	–	13.9 ± 10.4 μm	Physiological condition	(9)
Microvascular endothelial cells	Microvascular endothelial cells	F-actin or microtubules or both	180–400 nm	10–100 μm	Physiological condition	(19)
**Unidirectional mitochondrial transfer**						
Cardiomyocytes	MSCs	F-actin, or both F-actin and microtubules	760 ± 30 nm; or ~100 nm	31.66 ± 1.43 μm	Physiological condition; hypoxia	(11, 20–22)
Cardiomyocytes	Cardiac myofibroblasts	F-actin and microtubules	–		Hypoxia	(23)
Cardiomyocytes	Endothelial progenitor cells	–	50–800 nm	5–120 μm	Physiological condition	(8)
Stem cell	Neonatal cardiomyocytes	F-actin and microtubules	500–1,000 nm	80–100 μm	Physiological condition, lipopolysaccharide	(24)

*TNTs, tunneling nanotubes; HUVECs, umbilical vein endothelial cells; MSCs, mesenchymal stem cells*.

There are two different mechanisms involved in the formation of TNTs. **(1) Cell dislodgment mechanism**. Cells contact each other, then quickly migrate in opposite directions, retaining a thread of membrane between these two detached cells which finally develop into TNTs (9, 18). **(2) Actin-driven protrusion mechanism**. Filopodia-like membrane protrusions extend beyond the cell and elongate in an F-actin polymerization-dependent manner. Then the elongated protrusions connect with the target cells or other protrusions to form the TNTs (9, 12, 29). Unlike other cellular protrusions, these filopodia-like protrusions do not anchor to the substratum but suspend in the culture medium, which makes it possible for long-distance communication between cells (30).

M-Sec, also known as tumor necrosis factor α-inducible protein 2 (TNFαIP2), is reported as a key trigger of TNTs formation. Studies have shown that interaction of M-Sec with RalA can induce the assemble of exocyst complex and then initiate F-actin polymerization, while Cdc42 may be required for the extension process of TNTs (31). The expression of M-sec expression is regulated by many stimuli. Oxidative stress can activate p53, which in turn upregulates M-Sec expression by enhancing epidermal growth factor receptor expression or activating Akt/PI3K/mTOR pathway (32). Treatment of mesenchymal stem cells (MSCs) with TNF-α can increase M-Sec expression and trigger TNTs formation with cardiomyocytes *via* the NF-κB signaling pathway (15).

Some studies have demonstrated that gap junction protein connexin 43 (CX43) is necessary for TNTs formation and TNT-mediated intercellular mitochondrial transfer in non-cardiovascular systems (33–35). However, new evidence has shown that there is no Cx43 exists in the TNTs between cardiomyocytes and cardiac fibroblasts (9). Wang and coworkers have found that Cx43 only anchors at one end of TNT between two human umbilical vein endothelial cells (36). Since gap junctions do not allow the passage of large molecules (>1.2 kDa), Cx43 in the TNTs might only mediate intercellular electrical coupling but not cell-to-cell mitochondrial delivery (36). The role of Cx43 in the formation of TNTs and TNT-mediated mitochondrial transfer in the cardiovascular system still needs to be further explored.

Recently, mitochondrial Rho GTPase 1 (Miro1) has been reported to play a critical role in mediating mitochondrial movement along the TNTs (14, 15). Miro1 is a tail-anchored mitochondrial outer membrane protein. After combing with the adaptor protein TRAK1/2, Miro1 can recruit motor proteins (such as kinesin) and initiate microtubule-based mitochondrial movement (37). In a cardiomyocytes and cardiac myofibroblasts co-culture system, mitochondrial transport along microtubules in TNTs is mediated by KIF5B, which is a membrane of the kinesin superfamily (23). Recent studies have shown that Miro1 can also mediate actin-based mitochondrial transport *via* binding with motor protein Myo19 within individual mouse fibroblasts (38). However, whether Miro1 and Myo19 are involved in the mitochondrial movement along F-actin in TNTs still needs to be further investigated.

### Mitochondrial Transfer *via* Extracellular Vesicles

**Figure 2:**
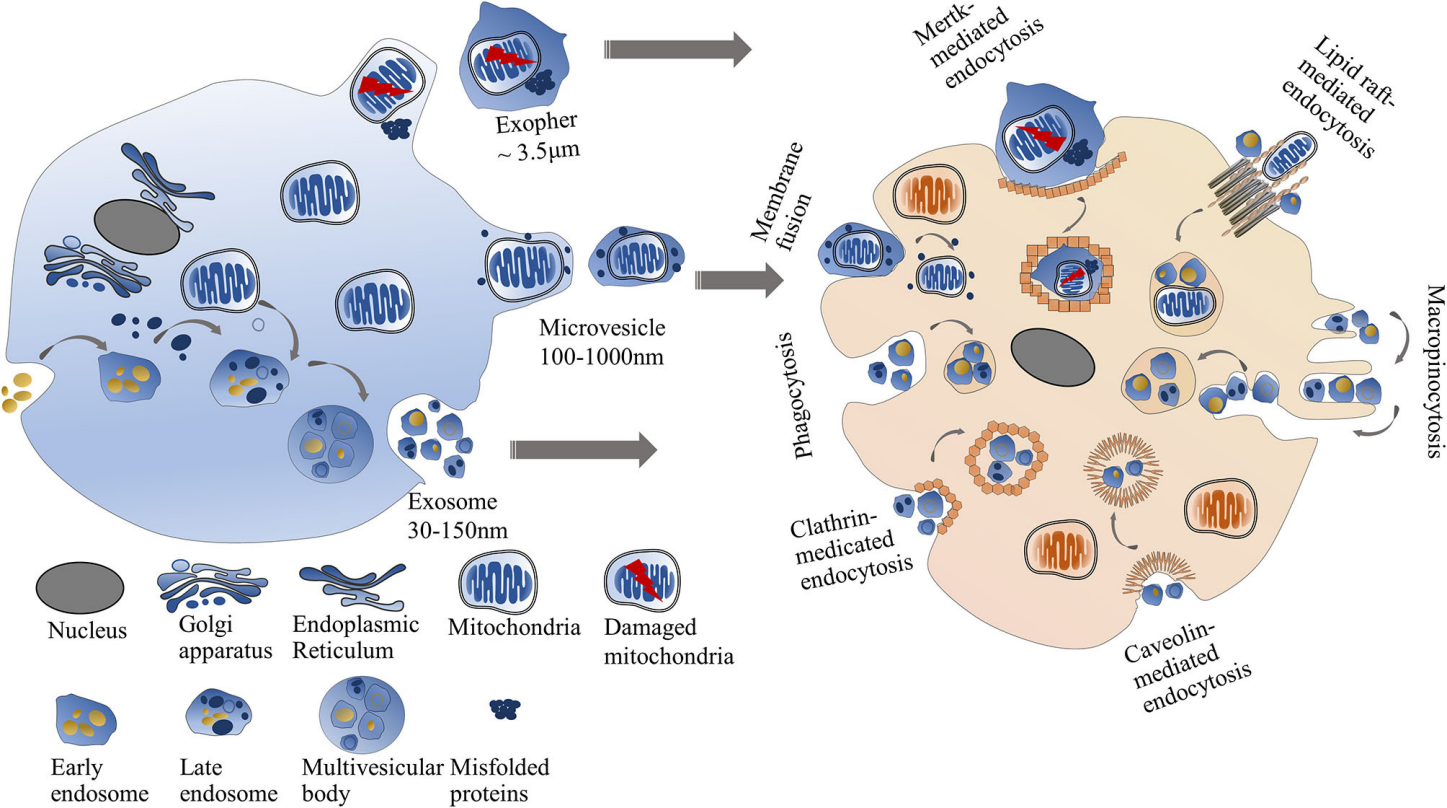
Mitochondrial transfer *via* EVs. The intercellular mitochondrial transfer can be mediated through EVs including exosomes, microvesicles, and exophers. Exosomes are smaller than microvesicles. Exophers are large membrane-surrounded microparticles usually containing damaged mitochondria and misfolded proteins. Exosomes or microvesicles can directly fuse with the recipient cell membrane or be engulfed by recipient cells through multiple pathways, including clathrin-dependent endocytosis, caveolin-mediated endocytosis, lipid raft-mediated endocytosis, phagocytosis, and micropinocytosis. Cardiac exophers can internalized into recipient cells *via* Mertk-mediated endocytosis.

Another pathway for cell-to-cell mitochondrial transfer is through EVs (Figure 2). The properties of EVs that transfer intact mitochondria or mitochondrial components in the cardiovascular system are listed in Table 2.

**Table 2:** Characteristics of mitochondrial transfer-related EVs in cardiovascular system.

**EVs**	**Donor cells**	**Recipient cells**	**Size**	**Compounds**	**Effect on recipient cells**	**References**
Exosomes	KSHV-infected HUVECs	Uninfected HUVECs	30–40 nm	mtDNA	Antiviral effect	(39)
Microvesicles	Healthy iCMs	Hypoxia-injured iCMs	98–677 nm	intact mitochondria	Improvement of intracellular energetics	(40)
Microvesicles	Lipopolysaccharide stimulated THP-1 monocytic cells	HUVECs	206.6 ± 89.8 nm	Intact mitochondria, and some mitochondrial components	Activation of inflammatory response	(41)
Exophers	Cardiomyocytes	Cardiac-resident macrophages	3.5 ± 0.1μm	Mitochondria	Preservation of metabolic stability	(42)

*EVs, extracellular vesicles; KSHV, Kaposi's sarcoma-associated herpesvirus; HUVECs, human umbilical vein endothelial cells; mtDNA, mitochondrial DNA; iCMs, induced pluripotent stem cell–derived cardiomyocytes*.

EVs are phospholipid membrane-bound microparticles released by cells. Exosomes and microvesicles are two major forms of EVs. Exosomes are small EVs (30–150 nm in diameter) that originated from the endosomal networks and are considered to deliver lipids, RNAs, and mitochondrial components (such as mtDNA). Microvesicles derived from cellular plasma membranes are larger than exosomes (100–1,000 nm in diameter) (43). Since mitochondria are elongated organelles with a diameter of 500–1,000 nm, the intact mitochondria more likely exist in the microvesicles but not in the exosomes (44). Many harmful stimuli, such as lipopolysaccharide, can induce endothelial cells to release EVs. Then the EVs are taken up by the recipient cells and cause inflammatory responses. The inflammatory responses might be due to the pro-inflammatory effect of mtDNA in the EVs (39, 41). On the contrary, hypoxia-injured cardiomyocytes can uptake the EVs containing respiratory-competent mitochondria to increase their rescue ability (40).

Recent studies have suggested that selective packaging of mitochondrial content into EVs depends on optic atrophy 1 (OPA1) and sorting nexin 9 (Snx9) proteins (45), but the exact mechanism is unclear. The formation of exosomes is initiated *via* membrane invagination to generate multivesicular late endosomes. Then the multivesicular late endosomes fuse with the plasma membrane, leading to the release of exosomes into the extracellular space. The biogenesis and release process of microvesicles is different from exosomes. Microvesicles are generated *via* membrane blebbing and then released into the extracellular environment by separating from the plasma membrane in Ca^2+^-dependent enzymatic machinery (46). Integrins on the surface of EVs have been widely reported as major regulators of anchoring EVs on recipient cells (47, 48). Once attaching the recipient cells, EVs can directly fuse with the recipient cell membrane or be engulfed by recipient cells through multiple pathways, including clathrin-dependent endocytosis, caveolin-mediated endocytosis, lipid raft-mediated endocytosis, phagocytosis, and micropinocytosis (49–51).

In 2020, a new type of mitochondria-containing EVs called exophers was discovered in hearts by Nicolas et al. (42). The structure of exophers from cardiac tissues is similar to that of neural exophers of *C. elegans*, which mainly contain misfolded proteins and damaged mitochondria (52). Different from the traditional EVs, cardiac exophers are large membrane-surrounded microparticles with an average diameter of 3.5 μm, which allows intact mitochondria to be packed in (42). The formation of cardiac exophers is motivated by the cardiac-specific autophagy mechanism. A large number of exophers extruded by cardiomyocytes can be engulfed by cardiac-resident macrophages *via* Mertk-mediated endocytosis. Such kind of crosstalk between cardiomyocytes and immune cells is required for the maintenance of mitochondrial fitness and cardiovascular health (42).

### Mitochondrial Transfer *via* Naked Mitochondria Extrusion

Many studies have shown that the intact respiratory competent mitochondria exist in healthy human and animal blood which might be released by resting or activated platelets (53, 54). Likewise, mitochondria can also be released into the environment in the form of naked organelles by many normal or abnormal cells beyond platelets (54). For example, extracellular mitochondria are found in the endothelial progenitor cells culture system under physiological conditions (55). Monocytic cells can extrude naked mitochondria after being attacked by lipopolysaccharide (41). It has been proven that intact cell-free mitochondria are released from platelets through an actin-dependent but microtubule-independent mechanism (56).

However, the uptake mechanism of cell-free mitochondria by recipient cells has not been fully clarified. A few previous reports have shown that MSCs engulf platelet-derived functional mitochondria through clathrin-mediated endocytosis and enhance their pro-angiogenic activity (49). Some evidence has demonstrated that autologous mitochondria can be internalized into cardiomyocytes through actin-dependent endocytosis. Neither caveola-mediated nor clathrin-mediated endocytosis is involved in the mitochondrial internalization into cardiomyocytes (57). It has been reported that H9C2 rat cardiomyocytes can recognize and engulf exogenous mitochondria released from human uterine endometrial gland-derived MSCs in a co-incubation system. The uptake of mitochondria by cardiomyocytes is mainly *via* micropinocytosis (58). During the mitochondrial internalization process, cells can discriminate intact mitochondria from other similar microparticles and only engulf mitochondria (59). So the internalization mechanism of naked mitochondria might be different according to the types of recipient cells and the origin of naked mitochondria.

### Mitochondrial Transfer *via* Other Pathways

Other pathways, such as cell fusion, are also found to be involved in intercellular mitochondrial transfer. In 2003, bone marrow-derived MSCs was reported to donate their mitochondria to cardiomyocytes through cell fusion. The cell fusion between MSCs and skeletal muscles is at a very low rate, suggesting that cell fusion is a kind of cell-specific machinery for cell-to-cell mitochondrial transfer (60).

## Fate of Donor Mitochondria in Recipient Cells

Studies have shown that most healthy donor mitochondria can successfully escape from the endo-lysosomal system after being transferred into damaged cardiomyocytes and quickly integrate into the host mitochondrial network (61, 62). The combination of donor and recipient mitochondria within cardiomyocytes is a transient event that lasts about 4 h (62). The mechanism of mitochondrial integration might involve dynamic movements of mitochondrial fusion and fission. Many studies have demonstrated that mitofusin 1 (Mfn1) and Mfn2 are necessary for the fusion of mitochondrial outer membrane, optic atrophy 1 (Opa1) is responsible for the fusion of mitochondrial inner membrane (63), and dynamin-related protein 1 (Drp1) is required for mitochondrial fission (64, 65). In non-cardiomyocytes, mitochondrial transplantation can enhance the expression of Mfn2 and Opa1 and decrease the level of Drp1, which results in mitochondrial fusion (66). In the co-culture system of iPS-derived cardiomyocytes and cardiac fibroblasts, the mitochondrial fusion of donor and recipient mitochondria might be more likely due to the high Mfn1 and Opa1 protein levels in mitochondria (62). A minority of donor mitochondria that cannot flee from lysosomes undergo degradation through autophagy (62). This phenomenon has been confirmed by Louwagie and coworkers, whose studies have shown that the number of lysosomes in the recipient cells elevates after 4 h of mitochondrial transfer, accompanied by a higher mitophagy of donor mitochondria and a lower mitophagy of host mitochondria (61).

On the contrary, the main function of transferring defective mitochondria from damaged cells to healthy cells is to ask for help. After that, these foreign mitochondria in recipient cells will eventually be trapped in the LC3B-labeled phagosomes and eliminated *via* mitophagy, which ensures the normal functions of recipient mitochondria (14).

Besides mitochondrial fusion, a structure called mitochondrial nanotunnels also allows the exchange of matrix between two individual mitochondria. The mitochondrial nanotunnels in cardiomyocytes are a thin double-membrane tunneling structure with 40–200 nm in diameter and 0.7–14 μm in length (67, 68). Mitochondrial components like mitochondrial DNA, proteins, lipids can freely diffuse through the mitochondrial nanotunnels. Although the rate of mitochondrial matrix exchange *via* mitochondrial nanotunnels is slower than that of mitochondrial fusion mode, it provides the possibility for long-range communication between two individual mitochondria (67, 68). Whether the mitochondrial nanotunnels participate in the communication of donor and recipient mitochondria still needs to be further explored.

## Role of Mitochondrial Transfer

### Role of Mitochondrial Transfer Under Physiological Conditions

The cell-to-cell mitochondrial transfer has been detected in the cardiovascular system under physiological conditions (Table 3). In 2005, the unidirectional mitochondrial transfer from neonatal cardiomyocytes to endothelial progenitor cells was observed for the first time by Koyanagi et al. (8). After receiving donor mitochondria, endothelial progenitor cells acquire a cardiomyocyte-like phenotype through reprogramming (8). Meanwhile, a bidirectional mitochondrial transfer has been detected between cardiac myocytes and MSCs in a co-culture system (22). Migration of mitochondria from MSCs into fully differentiated cardiomyocytes can reprogram the adult cardiomyocytes and regress them to a progenitor-like state (20). Likewise, the mitochondrial transfer from embryonic cardiomyocytes to MSCs initiates stem cells differentiation toward cardiac cells, which might be an essential mechanism of stem cell-based therapies for cardiovascular disorders (22). Studies have also shown that mitochondrial transfer between vascular smooth muscle cells and MSCs is required to promote stem cells proliferation (10). These results suggest that intercellular mitochondrial transfer might play an important role in the regulation of cardiovascular system development.

**Table 3:** Role of mitochondrial transfer under physiological and pathophysiological conditions.

**Role of mitochondrial transfer**	**Mechanism**	**References**
**Physiological condition**		
(1) Regulation of cardiovascular system development	• Reprograming the adult cardiomyocytes and endothelial progenitor cells • Promoting stem cells proliferation and differentiation toward cardiac cells	(8, 22) (10, 22)
(2) Maintaining normal cardiac homeostasis	• Clearance of dysfunctional mitochondria of cardiomyocytes by macrophages	(42)
**Pathophysiological condition**		
(1) Release of dysfunctional mitochondria to ask for help	• Mitochondria from damaged cardiomyocytes or endothelial cells acted as a danger signaling for stem cells	(14)
(2) Rescuing damaged cells by taking up functional mitochondria	• Improvement of mitochondrial biogenesis (elevating oxidative phosphorylation, reducing glycolysis, and increasing cellular ATP levels) • Enhancement of antioxidant capacity (overexpression of heme oxygenase-1) • Reduction of apoptosis (decrease of Bax/Bcl-2 ratio and the inhibition of caspase-3 activity)	(12, 15, 40, 57, 61, 69–71) (14, 15, 70) (11, 61)

Although cardiomyocytes account for about 70–85% of the adult myocardial tissue volume (72), non-myocytes in cardiac tissues are essential for heart health. Cardiomyocytes and cardiac fibroblasts are the two most abundant cell types in mammalian hearts. Recent studies have demonstrated that mitochondrial exchange between cardiomyocytes and fibroblasts is a distinct intercellular communication pattern, which might be indispensable for normal cardiac function (9). But the exact molecular mechanism remains unclear. In 2020, Nicolas-Avila and colleagues found that cardiomyocytes can eliminate their abnormal mitochondria by delivering them to heart-resident macrophages under physiological conditions. Harmful stimuli, such as ischemia or isoproterenol challenge, can enhance the efficiency of mitochondrial transfer and accelerate the clearance of dysfunctional mitochondria (42). The mitochondrial transfer from cardiomyocytes to macrophages is beneficial to maintain the mitochondrial fitness of cardiomyocytes, reduce the accumulation of pro-inflammatory material, and prevent the activation of inflammasome (42). Studies have also demonstrated a low mitochondrial transfer between heart-resident macrophages and other non-myocytes (such as endothelial cells), suggesting intercellular mitochondrial transfer within the heart has a highly cell-specific feature. These studies demonstrated that cell-to-cell mitochondrial transfer might be essential for maintaining normal cardiac homeostasis.

### Role of Mitochondrial Transfer Under Pathophysiological Conditions

Under pathophysiology conditions such as ischemic cardiomyopathy, damaged cells can not only release dysfunctional mitochondria to ask for help but also take up exogenous functional mitochondria to rescue their own mitochondria network (Table 3). The transfer of healthy mitochondria toward injured cells has multiple protective mechanisms include the following. **(1) Improvement of mitochondrial biogenesis**. The perturbation of mitochondrial biogenesis is known as the fundamental mechanism of cardiovascular diseases (6). Transfer of healthy mitochondria to the injured cardiomyocytes or endothelial cells can increase cellular ATP levels through elevating oxidative phosphorylation and tricarboxylic acid (TCA) cycle and reducing glycolysis (12, 15, 57, 61, 69). The improvement of mitochondrial biogenesis is due to the renewal of damaged mitochondrial DNA and increased expression of mitochondrial respiration-related protein through activation of peroxisome proliferator-activated receptor-gamma coactivator 1-alpha (PGC-1α)-mediated pathway (40, 57, 70, 71). It has been reported that this beneficial effect of the mitochondrial transfer can last for a long time (at least 28 days) in ischemic cardiomyocytes (73), which is in contrast to the short-term improvement of energy metabolism found in normal cardiomyocytes (74). **(2) Enhancement of antioxidant capacity**. Mitochondria are vital organelles that regulate redox balance *via* their pro-oxidant and antioxidant functions. Oxidative stress-induced injury is involved in the pathogenesis of many cardiovascular diseases, including atherosclerosis, myocardial ischemia-reperfusion injury, and hypertension (75–77). Inflammatory response, triggered by excessive ROS level, is also associated with vascular dysfunction in many pathophysiology conditions (78, 79). Recent studies have shown that the delivery of healthy mitochondria to cardiac cells or endothelial cells can protect them against oxidative damage (14, 70). Transplantation of MSCs to a doxorubicin-induced animal cardiomyopathy model also alleviates cardiac inflammation *via* mitochondrial transfer (15). The protective mechanism might be due to the overexpression of heme oxygenase-1, which has well-known properties of anti-oxidative and anti-inflammatory activities (14). **(3) Reduction of apoptosis**. Apoptosis is one of the most common patterns of programmed cell death in the cardiovascular system (80). Cardiomyocytes and endothelial cells are prone to apoptosis under various cellular stress (such as hypoxia, chemicals, and metabolic stress). Many studies have shown that transfer of healthy mitochondria to these injury cells can reduce apoptosis (11, 15, 18, 23, 69). The anti-apoptotic effect of mitochondrial transfer has been shown to have a gender-specific characteristic in pregestational diabetes mellitus-exposed offspring (61). The mechanism of mitochondrial transfer-induced anti-apoptosis might involve the decrease of Bax/Bcl-2 ratio and the inhibition of caspase-3 activity (11, 61).

In short, a series of studies implied the significance of mitochondrial transfer in the cardiovascular system. In physiological conditions, cardiac fibroblasts and cardiomyocytes show frequent intercellular communication through bidirectional mitochondrial transfer, which is critical in maintaining normal cardiac function (9). Although this phenomenon is observed in an *in vitro* model, whether it exists i*n vivo* has not been confirmed. Meanwhile, transferring distressed mitochondria to macrophages is also critical to the fitness of cardiomyocytes (42). It is undoubted that mitochondria containing the information of donor cells once internalized into recipient cells can trigger a cascade of response, which in turn acts on donor cells. For instance, cardiomyocytes and endothelium suffered ischemia/reperfusion injury deliver mitochondria as signals to MSCs to ask for help. After receiving mitochondria, MSCs enhance the biogenesis of mitochondria and promote the capacity of anti-apoptosis, then generously donate functional mitochondria to distressed cells (14).

## Therapeutic Strategies of Mitochondrial Transfer for Cardiovascular Diseases

**Figure 3:**
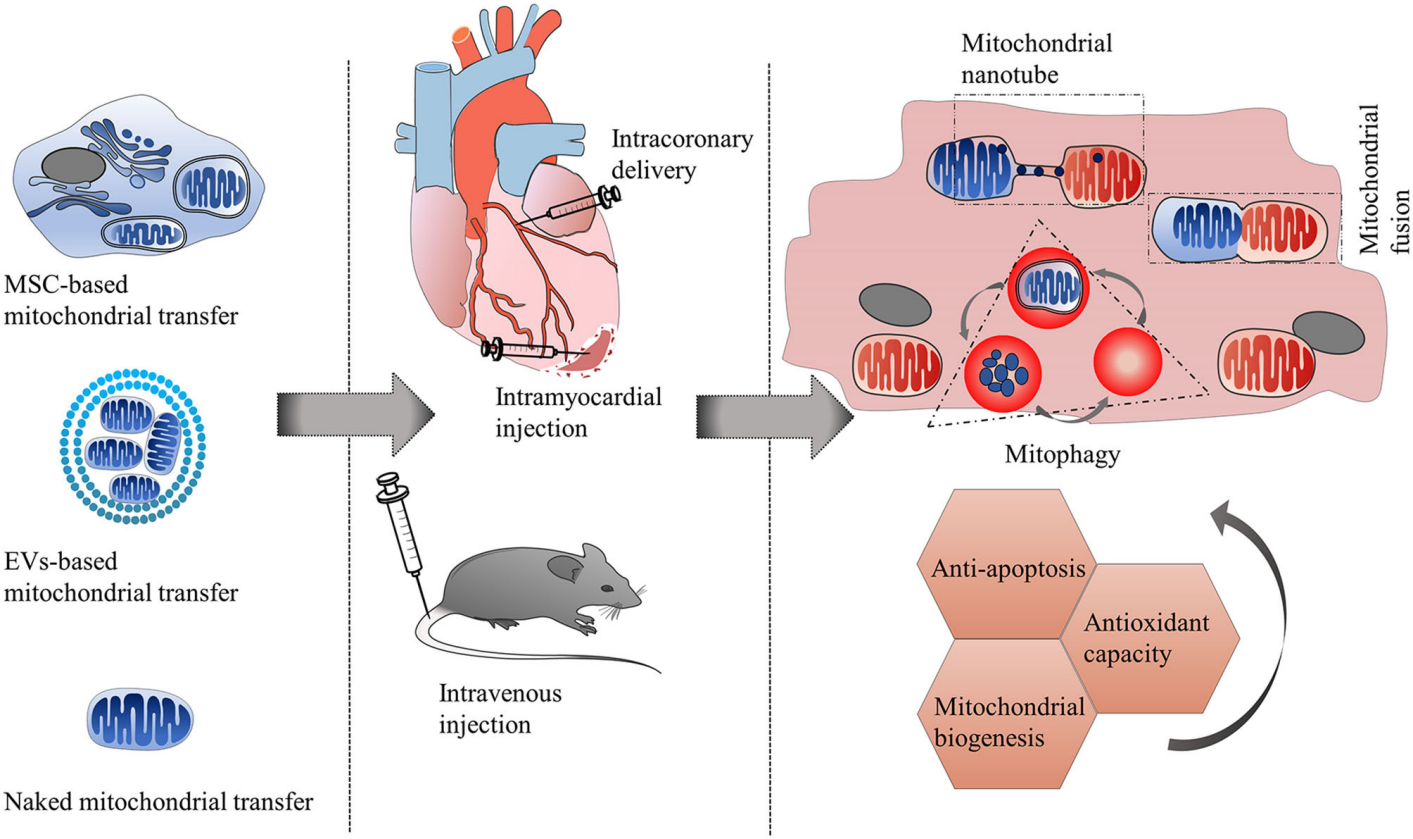
Therapeutic strategies of mitochondrial transfer for cardiovascular diseases. The most common methods of mitochondrial transplantation used for the treatment of cardiovascular diseases (such as ischemic cardiomyopathy, anthracycline-induced cardiomyopathy) are cell-mediated therapy and cell-free therapy (including naked mitochondria transplantation and EV-based transplantation). Routes of administration can be intramyocardial injection, intracoronary delivery, and intravenous injection. After approaching the recipient cells, exogenous mitochondria can integrate with recipient mitochondria through mitochondrial fusion and fission machinery, or be trapped by lysosomes and be autophagy degraded. Some donor mitochondria might only communicate with recipient mitochondria *via* mitochondrial nanotunnels, without undergoing mitochondrial fusion. The transfer of healthy mitochondria toward injured cells has multiple protective mechanisms including improvement of mitochondrial biogenesis, enhancement of antioxidant capacity and reduction of apoptosis.

Since transferring healthy mitochondria to damaged cells can alleviate injury and enhance the repairability of the target cells. Mitochondrial transplantation has been suggested as a promising therapeutic strategy for cardiovascular diseases. The most common methods of mitochondrial transplantation used for the treatment of cardiovascular diseases are cell-mediated therapy and cell-free therapy (including naked mitochondria transplantation and EV-based transplantation) (Figure 3 and Table 4).

**Table 4:** Summary of mitochondrial transfer strategies for cardiovascular diseases.

	**Cell-based mitochondrial transplantation**	**EV-based mitochondrial transplantation**	**Naked mitochondrial transplantation**
Origins	MSCs and progenitor cells	MSCs-derived EVs	Mitochondria isolated from healthy cardiac or skeletal muscle
Application	Ischemia-reperfusion injury, and anthracycline-induced cardiomyopathy	Ischemia-reperfusion injury	Right heart failure, ischemia-reperfusion injury, and ischemia/reperfusion injury of diabetic heart
Route of administration	Intramyocardial injection	Intracoronary or intravenous injection	Intramyocardial, intracoronary, or intravenous injection
Major outcome	Improving cardiac function Alleviating left ventricular dilatation Reducing myocardial fibrosis	Improving myocardial contractility Preventing left ventricular remodeling	Decreasing infarct size Improving ventricular function Enhancing coronary blood flow Delaying the progression of right heart failure
Advantages	Abundant sources Good quality and integrity of mitochondria	High stability of mitochondria No risk of microvascular obstruction No risk of cardiac arrhythmia	No risk of autoimmune response No risk of microvascular obstruction No risk of cardiac arrhythmia Without intramyocardial hematoma
Disadvantages	Undesired differentiation Cardiac arrhythmia Microcirculation occlusion Difference in mitochondrial transfer capacity and effectiveness due to origins of MSCs	Heterogeneity of EVs' cargo content due to different cellular origins and isolation methods	Lower stability than EV-coated mitochondia Low yields of good-quality mitochondria Limited viability of transferred mitochondria For intramyocardial injection: (1) multiple injections are required; (2) The need for thoracotomy prior to the intramyocardial injection; (3) lower mitochondrial internalization (3~7%) than that of intracoronary injection; (4) clusters found in intramyocardial injection site For intravenous injection: lack of tissue-specific delivery
Reference	(15, 81–84)	(40)	(62, 71, 73, 85–95)

*EV, extracellular vesicle; MSCs, mesenchymal stem cells*.

### Cell-Based Mitochondrial Transplantation

MSCs and progenitor cells have been recognized as the preferred mitochondria donors for the treatment of cardiovascular diseases due to their abundant sources and high mitochondrial respiratory activity. Animal experiments and clinical trials have shown that transplantation of MSCs can successfully repair injured myocardium in ischemic cardiomyopathy (81–84). The protective mechanism of MSCs therapy has been proven mainly *via* mitochondrial transfer in *vitro* or in *vivo* studies. For example, MSCs can prevent vascular endothelial cells injury in an ischemia-reperfusion model by enhancing aerobic respiration and reducing apoptosis through transferring mitochondria to endothelial cells (12). Mitochondria transferring from MSCs can protect cardiomyocytes against oxidative stress-induced injury by improving mitochondrial respiration function (11, 14, 15, 17, 70). MSCs transplantation has also been reported to reduce myocardial fibrosis, alleviate left ventricular dilatation, and improve cardiac function in an animal model of anthracycline-induced cardiomyopathy through mitochondrial transfer (15).

MSCs used in clinical trials can be originated from widely various tissues. Paliwal and coworkers have reported that the MSCs derived from pulp and Wharton's jelly have lower mitochondrial transfer abilities but higher mitochondrial respiration capacities than those of MSCs from bone marrow and adipose (70). Compared with bone marrow-derived MSCs, human-induced pluripotent stem cell-derived MSCs have higher efficiency of mitochondrial transfer due to their higher expression of Miro1 and TNFαIP2 (15). So the difference in mitochondrial transfer capacity and effectiveness of these tissue-specific MSCs need to be considered in cell-based therapy for cardiovascular diseases.

Whether the beneficial effect of MSCs transplantation is mainly due to mitochondrial transfer remains controversial. A few previous studies have reported that MSCs can rescue damaged cells through paracrine mechanisms (96). However, many in *vitro* studies have proven that the cardiovascular protective effect of stem cell-based therapy is mainly dependent on the transfer of functional mitochondria rather than the secretion of paracrine factors (12, 15). Since MSCs have limited trans-differentiation abilities into cardiomyocytes or other vascular components *in vivo*, it seems that the protective effect of MSCs transplantation is also unlikely due to their differentiation capacity (97). However, recent studies have shown that the emergence of many safety issues such as undesired differentiation, pro-arrhythmia, and microcirculation occlusion limit the clinical use of stem cell-based therapy (98–100).

### Naked Mitochondria Transplantation

Naked mitochondrial transplantation refers to the transplantation of isolated and uncoated mitochondria to the injured tissues through circulation delivery or local injection (85, 86). The transplantation of naked mitochondria for the treatment of cardiovascular diseases can be traced back to 2009 when McCully's laboratory at Harvard demonstrated for the first time that intramyocardial injection of respiration-competent mitochondria could reduce infarct size and promote postischemic functional recovery in an animal model of heart ischemia-reperfusion injury (85). The optimal dose of mitochondria needed for efficiently protecting against ischemia-reperfusion injury ranges from 2 × 10^5^ to 2 × 10^8^ per gram wet weight (62, 71, 73, 87). Autologous mitochondria isolated from healthy cardiac or skeletal muscle are the dominant sources of donor mitochondria (85, 87, 101). The uptake of autologous mitochondria by cardiomyocytes is usually within minutes *via* internalization (62, 73). Exogenous mitochondria can also enter into cardiomyocytes without being cleared by lysosomes or autophagosomes. However, it takes more than 8 h for cardiomyocytes to engulf the xenogeneic mitochondria (73). In 2017, the first clinical use of mitochondrial transplantation was performed in five pediatric patients who suffered from cardiac ischemia-reperfusion injury. Four in five patients who accepted intramyocardial injection of autologous mitochondria have shown an improvement in ventricular function without any short-term side effects (such as arrhythmia, mitochondrial autoimmune response, and intramyocardial hematoma) (101).

Although intramyocardial injection of mitochondria has been proven efficient and safe in the treatment of myocardial ischemic disease, there are still a few limitations. For example, only a small number of donor mitochondria is allowed to inject within the myocardium per shot, and the percentage of mitochondrial internalization at each injection site is also pretty low (about 3–7%) (71, 73, 87). So multiple injections are required to ensure the extensive distribution of donor mitochondria throughout the ischemic heart, which increases the difficulty of operation. In addition, the need for thoracotomy prior to the intramyocardial injection may also be a huge obstacle limiting the clinical application of mitochondrial transplantation for potential patients (101).

Since 2016, researchers have begun to test the feasibility of intracoronary delivery as an alternative mitochondrial transplantation method (88). Both autologous and exogenous mitochondria can rapidly spread throughout the whole heart within 10 min of coronary perfusion rather than occur in clusters as found in intramyocardial injection. Furthermore, intracoronary delivery also results in a higher mitochondrial internalization into cardiomyocytes (~23%) than intramyocardial injection (88). In animal models of regional ischemia-reperfusion injury, both preischemic or postischemic intracoronary injection of autologous mitochondria can decrease infarct size, enhance coronary blood flow, and increase cardiac function (88–91). Intracoronary injection of healthy mitochondria also has powerful cardiac protection against globally ischemic injury of donor hearts and global ischemia/reperfusion injury of diabetic heart (88, 92–94). No signs of microvascular obstruction and cardiac arrhythmia are observed after intracoronary injection of mitochondria (91). The safety and efficacy of intracoronary delivery of mitochondria make it a promising treatment for myocardial infarction through percutaneous coronary intervention.

Considering the clinical use of mitochondrial transplantation, intravenous injection of mitochondria might be more feasible than intramyocardial and intracoronary administration. Intravenous delivery of mitochondria has been used as a promising therapeutic method for fatty liver and Parkinson's disease (102, 103). Recent studies have shown that intravenous injection of viable exogenous mitochondria for 3 weeks can improve right ventricular function in an animal model of pulmonary hypertension (86). After systemic administration, the mitochondria are observed in various tissues, including heart (102, 103). The stability of naked mitochondria in the bloodstream and tissue-specific delivery may be the key factors for successful therapy in ischemic or non-ischemic cardiovascular disorders.

Besides ischemic heart injury, recent studies have also found that mitochondrial transplantation can delay the progression of right heart failure (86, 95) and improve myocardium metabolism of offspring born to diabetic mothers (61). Although studies have confirmed the efficiency of naked mitochondrial transplantation, how to obtain high yields of good-quality mitochondria is still a challenge. Enhancing the efficiency of mitochondrial internalization into target cells and maintaining the viability of transferred mitochondria are also crucial problems to assure the efficient clinical application of naked mitochondria transplantation.

### EV-Based Mitochondrial Transplantation

Another method of cell-free therapy is mitochondria-rich EVs transplantation. EVs have been recognized as a powerful platform for mitochondrial delivery (40, 104, 105). MSCs from different tissues are the main sources of EVs (106). Although many studies have proven that MSCs-derived EVs can serve as a potential therapy for the treatment of cardiovascular disease, whether the protective effect is mainly dependent on their mitochondria cargo is unclear (107). In 2021, Ikeda's laboratory at Stanford isolated some mitochondria-rich EVs from human-induced pluripotent stem cell-derived cardiomyocytes. The diameter of these EVs ranges from 98 to 677 nm (40). The outer lipid bilayer of EVs usually serves as a security guard that keeps the mitochondria maintaining their morphological and functional integrity. The mitochondria encapsulated within EVs are found more stable than naked mitochondria under extracellular environmental stress, such as calcium overload and oxidative stress (40). In *vitro* and in *vivo* studies have demonstrated that transplantation of mitochondria-rich EVs can restore intracellular bioenergetics, prevent post-ischemic left ventricular remodeling, and improve myocardial contractility (40). Since the diameters of MSC-derived EVs are usually <10 μm (108), intravenous or intracoronary injection of EVs has no risk of microvascular obstruction. The intramyocardial injection of mitochondria-rich EVs into the peri-infarct region does not induce cardiac arrhythmia (40), which supports the opinion of Adamiak and coworkers that MSC-derived EVs are safer than MSCs (109). The cargo content of EVs mainly includes mitochondria and their components, nucleic acids, lipids, and proteins, which can be altered according to their cellular origins and isolation methods (107). The complex composition of different EVs makes them have distinctive mechanisms and effects on various diseases. Ikeda and coworkers have found that the beneficial effect of mitochondria-rich EVs transplantation on myocardial ischemia-reperfusion injury was not only due to mitochondria cargo but also due to non-mitochondrial cargo (40). In order to reduce the heterogeneity of EVs and guarantee the therapeutic effect, it is necessary to set up a standardized EV isolation protocol. Meanwhile, how to improve the targeting specificity of EVs is also an issue needed to be further investigated (110).

In conclusion, many in *vivo* studies have proven the effectiveness of mitochondrial transplantation in the treatment of different cardiovascular diseases. Ischemia/reperfusion injury is one of the most common diseases in the cardiovascular system. Researchers delivered mitochondria to rescue damaged cardiac tissue through different administrational routes in various species (14, 40, 69, 73). Comfortingly, all of these studies have confirmed the significant improvement of cardiac function after mitochondrial transplantation. In a clinical trial, autologous mitochondria from skeletal muscle were injected into the damaged cardiomyocytes of pediatric patients suffering ischemia/reperfusion injury, which effectively promote the recovery of postischemic myocardium without adverse short-term complications (101). Functional mitochondrial delivery has been used for some other cardiovascular diseases (including heart failure, anthracycline-induced cardiomyopathy, pregestational diabetes-induced cardiac malfunction, heart transplantation) and to some extent improve the prognosis (15, 61, 70, 94).

## Ethical Issues

In 2015, the United Kingdom became the first country in the world to legislate and permit the clinical use of mitochondrial donation technology (111). However, there are still controversies about the ethical issues of the mitochondrial transfer strategy. **(1) Mitochondrial-nuclear incompatibility**. Studies have shown that the efficiency of cellular energy metabolism depends on about 2000 mitochondrial proteins. Most of these proteins are encoded by the nuclear genome, and only 13 proteins are encoded by the mitochondrial genome (112). So the compatibility of donor mitochondria and recipient cell nuclei is critical for the normal mitochondrial respiratory function of recipient cells (113–115). Many researchers have suggested that mitochondria originated only from the same cells or species are the ideal donors for reducing mitochondria-nuclear incompatibility and ensuring successful mitochondrial transfer (116, 117). **(2) Transmission of detrimental mutation**. The mutation rate of mtDNA is significantly higher than that of nuclear DNA due to a lack of histone protection and less efficient repairability (118). Meanwhile, studies have shown that mitochondrial fragmentation of donor mitochondria increases when using the standard mitochondrial isolation methods. And elevated mitochondrial fission is closely related to mtDNA abnormalities (119, 120). The mutated mtDNA of donor mitochondria can be transmitted to the recipient cells during intercellular mitochondrial transfer. When the accumulation of mutated mtDNA exceeds the threshold, cellular dysfunction, and abnormal morphology will occur (121). Such detrimental effects on recipient cells might be unpredictable due to intra- and inter-cellular mitochondrial heterogeneity (122). Therefore, the establishment of ethical guidelines is a prerequisite for ensuring the safe application of mitochondrial transfer strategies in the treatment of cardiovascular diseases.

## Discussion and Future Perspectives

Mitochondrial dysfunction plays a crucial role in the development and progression of cardiovascular disorders, which provides the possibility for mitochondrial transfer as an effective therapeutic strategy in the treatment of cardiovascular diseases. With the emergence of new technologies, trends in mitochondrial transplantation therapeutics are changing from cell-based to cell-free therapy. EVs-based mitochondrial delivery is considered more promising than naked mitochondria transplantation in the treatment of cardiovascular diseases, but it still has some limitations. Recently, some researchers developed a new delivery system by artificially encapsulating isolated mitochondria with some biocompatible polymers (such as dextran triphenylphosphonium complexes, transactivator of transcription dextran complexes) (69, 123). The polymer-coated delivery system, which almost exclusively contains mitochondria, is considered better than the EV-based delivery system. Meanwhile, the polymer-coated mitochondria have a higher transfer efficiency and a more powerful rescue capability than those of naked mitochondria, suggesting that they might become a more feasible and promising strategic alternative for mitochondrial transplantation in the future (69, 123). Although many preclinical experiments have proven the advantages of mitochondrial delivery in treating cardiovascular diseases, there are still a few technical challenges and ethical issues that need to be resolved (124). The efficiency and safety of mitochondrial transplantation in treating cardiovascular diseases still need to be further evaluated before conducting clinical trials.

## Author Contributions

JC wrote the manuscript and prepared the tables. JZ wrote the manuscript and made the figures. L-lW and Y-yC edited the manuscript. All authors contributed to the article and approved the submitted version.

## Funding

This work was supported by the National Natural Science Foundation of China (Grant Numbers: 81871541 and 81471837).

## Conflict of Interest

The authors declare that the research was conducted in the absence of any commercial or financial relationships that could be construed as a potential conflict of interest.

## Publisher's Note

All claims expressed in this article are solely those of the authors and do not necessarily represent those of their affiliated organizations, or those of the publisher, the editors and the reviewers. Any product that may be evaluated in this article, or claim that may be made by its manufacturer, is not guaranteed or endorsed by the publisher.

## References

[1] Zhang Y Murugesan P Huang K Cai H. NADPH oxidases and oxidase crosstalk in cardiovascular diseases: novel therapeutic targets. Nat Rev Cardiol. (2020) 17:170–94. 10.1038/s41569-019-0260-8 31591535 PMC7880919

[2] Sun HJ Wu ZY Nie XW Bian JS. Role of endothelial dysfunction in cardiovascular diseases: the link between inflammation and hydrogen sulfide. Front Pharmacol. (2019) 10:1568. 10.3389/fphar.2019.01568 32038245 PMC6985156

[3] Dey S DeMazumder D Sidor A Foster DB O'Rourke B. Mitochondrial ROS drive sudden cardiac death and chronic proteome remodeling in heart failure. Circ Res. (2018) 123:356–71. 10.1161/CIRCRESAHA.118.312708 29898892 PMC6057154

[4] Zhou B Tian R. Mitochondrial dysfunction in pathophysiology of heart failure. J Clin Invest. (2018) 128:3716–26. 10.1172/JCI120849 30124471 PMC6118589

[5] Bonora M Wieckowski MR Sinclair DA Kroemer G Pinton P Galluzzi L. Targeting mitochondria for cardiovascular disorders: therapeutic potential and obstacles. Nat Rev Cardiol. (2019) 16:33–55. 10.1038/s41569-018-0074-0 30177752 PMC6349394

[6] Poznyak AV Ivanova EA Sobenin IA Yet SF Orekhov AN. The role of mitochondria in cardiovascular diseases. Biology. (2020) 9:137. 10.3390/biology9060137 32630516 PMC7344641

[7] Spees JL Olson SD Whitney MJ Prockop DJ. Mitochondrial transfer between cells can rescue aerobic respiration. Proc Natl Acad Sci USA. (2006) 103:1283–8. 10.1073/pnas.0510511103 16432190 PMC1345715

[8] Koyanagi M Brandes RP Haendeler J Zeiher AM Dimmeler S. Cell-to-cell connection of endothelial progenitor cells with cardiac myocytes by nanotubes: a novel mechanism for cell fate changes? Circ Res. (2005) 96:1039–41. 10.1161/01.RES.0000168650.23479.0c 15879310

[9] He K Shi X Zhang X Dang S Ma X Liu F. Long-distance intercellular connectivity between cardiomyocytes and cardiofibroblasts mediated by membrane nanotubes. Cardiovasc Res. (2011) 92:39–47. 10.1093/cvr/cvr189 21719573

[10] Vallabhaneni KC Haller H Dumler I. Vascular smooth muscle cells initiate proliferation of mesenchymal stem cells by mitochondrial transfer via tunneling nanotubes. Stem Cells Dev. (2012) 21:3104–13. 10.1089/scd.2011.0691 22676452 PMC3495124

[11] Han H Hu J Yan Q Zhu J Zhu Z Chen Y. Bone marrow-derived mesenchymal stem cells rescue injured H9c2 cells via transferring intact mitochondria through tunneling nanotubes in an *in vitro* simulated ischemia/reperfusion model. Mol Med Rep. (2016) 13:1517–24. 10.3892/mmr.2015.4726 26718099 PMC4732861

[12] Liu K Ji K Guo L Wu W Lu H Shan P. Mesenchymal stem cells rescue injured endothelial cells in an *in vitro* ischemia-reperfusion model via tunneling nanotube like structure-mediated mitochondrial transfer. Microvasc Res. (2014) 92:10–8. 10.1016/j.mvr.2014.01.008 24486322

[13] Davis CH Kim KY Bushong EA Mills EA Boassa D Shih T. Transcellular degradation of axonal mitochondria. Proc Natl Acad Sci USA. (2014) 111:9633–8. 10.1073/pnas.1404651111 24979790 PMC4084443

[14] Mahrouf-Yorgov M Augeul L Da Silva CC Jourdan M Rigolet M Manin S. Mesenchymal stem cells sense mitochondria released from damaged cells as danger signals to activate their rescue properties. Cell Death Differ. (2017) 24:1224–38. 10.1038/cdd.2017.51 28524859 PMC5520168

[15] Zhang Y Yu Z Jiang D Liang X Liao S Zhang Z. iPSC-MSCs with high intrinsic MIRO1 and sensitivity to TNF-alpha yield efficacious mitochondrial transfer to rescue anthracycline-induced cardiomyopathy. Stem Cell Rep. (2016) 7:749–63. 10.1016/j.stemcr.2016.08.009 27641650 PMC5063626

[16] Rustom A Saffrich R Markovic I Walther P Gerdes HH. Nanotubular highways for intercellular organelle transport. Science. (2004) 303:1007–10. 10.1126/science.1093133 14963329

[17] Cselenyak A Pankotai E Horvath EM Kiss L Lacza Z. Mesenchymal stem cells rescue cardiomyoblasts from cell death in an *in vitro* ischemia model via direct cell-to-cell connections. BMC Cell Biol. (2010) 11:29. 10.1186/1471-2121-11-29 20406471 PMC2869333

[18] Feng Y Zhu R Shen J Wu J Lu W Zhang J. Human bone marrow mesenchymal stem cells rescue endothelial cells experiencing chemotherapy stress by mitochondrial transfer via tunneling nanotubes. Stem Cells Dev. (2019) 28:674–82. 10.1089/scd.2018.0248 30808254

[19] Astanina K Koch M Jungst C Zumbusch A Kiemer AK. Lipid droplets as a novel cargo of tunnelling nanotubes in endothelial cells. Sci Rep. (2015) 5:11453. 10.1038/srep11453 26095213 PMC4476149

[20] Acquistapace A Bru T Lesault PF Figeac F Coudert AE le Coz O. Human mesenchymal stem cells reprogram adult cardiomyocytes toward a progenitor-like state through partial cell fusion and mitochondria transfer. Stem Cells. (2011) 29:812–24. 10.1002/stem.632 21433223 PMC3346716

[21] Zhang J Zhang J Zhao L Xin Y Liu S Cui W. Differential roles of microtubules in the two formation stages of membrane nanotubes between human mesenchymal stem cells and neonatal mouse cardiomyocytes. Biochem Biophys Res Commun. (2019) 512:441–7. 10.1016/j.bbrc.2019.03.075 30904163

[22] Plotnikov EY Khryapenkova TG Vasileva AK Marey MV Galkina SI Isaev NK. Cell-to-cell cross-talk between mesenchymal stem cells and cardiomyocytes in co-culture. J Cell Mol Med. (2008) 12:1622–31. 10.1111/j.1582-4934.2007.00205.x 18088382 PMC3918078

[23] Shen J Zhang JH Xiao H Wu JM He KM Lv ZZ. Mitochondria are transported along microtubules in membrane nanotubes to rescue distressed cardiomyocytes from apoptosis. Cell Death Dis. (2018) 9:81. 10.1038/s41419-017-0145-x 29362447 PMC5833423

[24] Yang H Borg TK Ma Z Xu M Wetzel G Saraf LV. Biochip-based study of unidirectional mitochondrial transfer from stem cells to myocytes via tunneling nanotubes. Biofabrication. (2016) 8:015012. 10.1088/1758-5090/8/1/015012 26844857

[25] MacAskill AF Kittler JT. Control of mitochondrial transport and localization in neurons. Trends Cell Biol. (2010) 20:102–12. 10.1016/j.tcb.2009.11.002 20006503

[26] Dupont M Souriant S Lugo-Villarino G Maridonneau-Parini I Verollet C. Tunneling nanotubes: intimate communication between myeloid cells. Front Immunol. (2018) 9:43. 10.3389/fimmu.2018.00043 29422895 PMC5788888

[27] Li RF Zhang W Man QW Zhao YF Zhao Y. Tunneling nanotubes mediate intercellular communication between endothelial progenitor cells and osteoclast precursors. J Mol Histol. (2019) 50:483–91. 10.1007/s10735-019-09842-y 31463584

[28] Panasiuk M Rychlowski M Derewonko N Bienkowska-Szewczyk K. Tunneling nanotubes as a novel route of cell-to-cell spread of herpesviruses. J Virol. (2018) 92:e00090–18. 10.1128/JVI.00090-18 29491165 PMC5923070

[29] Qin Y Jiang X Yang Q Zhao J Zhou Q Zhou Y. The functions, methods, and mobility of mitochondrial transfer between cells. Front Oncol. (2021) 11:672781. 10.3389/fonc.2021.672781 34041035 PMC8141658

[30] Gerdes HH Bukoreshtliev NV Barroso JF. Tunneling nanotubes: a new route for the exchange of components between animal cells. FEBS Lett. (2007) 581:2194–201. 10.1016/j.febslet.2007.03.071 17433307

[31] Hase K Kimura S Takatsu H Ohmae M Kawano S Kitamura H. M-Sec promotes membrane nanotube formation by interacting with Ral and the exocyst complex. Nat Cell Biol. (2009) 11:1427–32. 10.1038/ncb1990 19935652

[32] Wang Y Cui J Sun X Zhang Y. Tunneling-nanotube development in astrocytes depends on p53 activation. Cell Death Differ. (2011) 18:732–42. 10.1038/cdd.2010.147 21113142 PMC3131904

[33] Osswald M Jung E Sahm F Solecki G Venkataramani V Blaes J. Brain tumour cells interconnect to a functional and resistant network. Nature. (2015) 528:93–8. 10.1038/nature16071 26536111

[34] Yao Y Fan XL Jiang D Zhang Y Li X Xu ZB. Connexin 43-mediated mitochondrial transfer of iPSC-MSCs alleviates asthma inflammation. Stem Cell Reports. (2018) 11:1120–35. 10.1016/j.stemcr.2018.09.012 30344008 PMC6234920

[35] Tishchenko A Azorin DD Vidal-Brime L Munoz MJ Arenas PJ Pearce C. Cx43 and associated cell signaling pathways regulate tunneling nanotubes in breast cancer cells. Cancers. (2020) 12:2798. 10.3390/cancers12102798 33003486 PMC7601615

[36] Wang X Veruki ML Bukoreshtliev NV Hartveit E Gerdes HH. Animal cells connected by nanotubes can be electrically coupled through interposed gap-junction channels. Proc Natl Acad Sci USA. (2010) 107:17194–9. 10.1073/pnas.1006785107 20855598 PMC2951457

[37] Kassab S Albalawi Z Daghistani H Kitmitto A. Mitochondrial arrest on the microtubule highway-a feature of heart failure and diabetic cardiomyopathy? Front Cardiovasc Med. (2021) 8:689101. 10.3389/fcvm.2021.689101 34277734 PMC8282893

[38] Lopez-Domenech G Covill-Cooke C Ivankovic D Halff EF Sheehan DF Norkett R. Miro proteins coordinate microtubule- and actin-dependent mitochondrial transport and distribution. EMBO J. (2018) 37:321–36. 10.15252/embj.201696380 29311115 PMC5793800

[39] Jeon H Lee J Lee S Kang SK Park SJ Yoo SM. Extracellular vesicles from KSHV-infected cells stimulate antiviral immune response through mitochondrial DNA. Front Immunol. (2019) 10:876. 10.3389/fimmu.2019.00876 31068945 PMC6491682

[40] Ikeda G Santoso MR Tada Y Li AM Vaskova E Jung JH. Mitochondria-rich extracellular vesicles from autologous stem cell-derived cardiomyocytes restore energetics of ischemic myocardium. J Am Coll Cardiol. (2021) 77:1073–88. 10.1016/j.jacc.2020.12.060 33632482 PMC8626617

[41] Puhm F Afonyushkin T Resch U Obermayer G Rohde M Penz T. Mitochondria are a subset of extracellular vesicles released by activated monocytes and induce type I IFN and TNF responses in endothelial cells. Circ Res. (2019) 125:43–52. 10.1161/CIRCRESAHA.118.314601 31219742

[42] Nicolas-Avila JA Lechuga-Vieco AV Esteban-Martinez L Sanchez-Diaz M Diaz-Garcia E Santiago DJ. A network of macrophages supports mitochondrial homeostasis in the heart. Cell. (2020) 183:94–109 e23. 10.1016/j.cell.2020.08.031 32937105

[43] French KC Antonyak MA Cerione RA. Extracellular vesicle docking at the cellular port: extracellular vesicle binding and uptake. Semin Cell Dev Biol. (2017) 67:48–55. 10.1016/j.semcdb.2017.01.002 28104520 PMC5484727

[44] Ratajczak MZ Ratajczak J. Horizontal transfer of RNA and proteins between cells by extracellular microvesicles: 14 years later. Clin Transl Med. (2016) 5:7. 10.1186/s40169-016-0087-4 26943717 PMC4779088

[45] Todkar K Chikhi L Desjardins V El-Mortada F Pepin G Germain M. Selective packaging of mitochondrial proteins into extracellular vesicles prevents the release of mitochondrial DAMPs. Nat Commun. (2021) 12:1971. 10.1038/s41467-021-21984-w 33785738 PMC8009912

[46] Guse AH. Second messenger function and the structure-activity relationship of cyclic adenosine diphosphoribose (cADPR). FEBS J. (2005) 272:4590–7. 10.1111/j.1742-4658.2005.04863.x 16156781

[47] Pang A Cui Y Chen Y Cheng N Delaney MK Gu M. Shear-induced integrin signaling in platelet phosphatidylserine exposure, microvesicle release, and coagulation. Blood. (2018) 132:533–43. 10.1182/blood-2017-05-785253 29853537 PMC6073322

[48] Tang TT Lv LL Wang B Cao JY Feng Y Li ZL. Employing macrophage-derived microvesicle for kidney-targeted delivery of dexamethasone: an efficient therapeutic strategy against renal inflammation and fibrosis. Theranostics. (2019) 9:4740–55. 10.7150/thno.33520 31367254 PMC6643445

[49] Levoux J Prola A Lafuste P Gervais M Chevallier N Koumaiha Z. Platelets facilitate the wound-healing capability of mesenchymal stem cells by mitochondrial transfer and metabolic reprogramming. Cell Metab. (2021) 33:283–99 e9. 10.1016/j.cmet.2020.12.006 33400911

[50] van Niel G D'Angelo G Raposo G. Shedding light on the cell biology of extracellular vesicles. Nat Rev Mol Cell Biol. (2018) 19:213–28. 10.1038/nrm.2017.125 29339798

[51] Zhang Y Tan J Miao Y Zhang Q. The effect of extracellular vesicles on the regulation of mitochondria under hypoxia. Cell Death Dis. (2021) 12:358. 10.1038/s41419-021-03640-9 33824273 PMC8024302

[52] Melentijevic I Toth ML Arnold ML Guasp RJ Harinath G Nguyen KC. *C. elegans* neurons jettison protein aggregates and mitochondria under neurotoxic stress. Nature. (2017) 542:367–71. 10.1038/nature21362 28178240 PMC5336134

[53] Stephens OR Grant D Frimel M Wanner N Yin M Willard B. Characterization and origins of cell-free mitochondria in healthy murine and human blood. Mitochondrion. (2020) 54:102–12. 10.1016/j.mito.2020.08.002 32781153 PMC7508808

[54] Al Amir Dache Z Otandault A Tanos R Pastor B Meddeb R Sanchez C. Blood contains circulating cell-free respiratory competent mitochondria. Faseb J. (2020) 34:3616–30. 10.1096/fj.201901917RR 31957088

[55] Hayakawa K Chan SJ Mandeville ET Park JH Bruzzese M Montaner J. Protective effects of endothelial progenitor cell-derived extracellular mitochondria in brain endothelium. Stem Cells. (2018) 36:1404–10. 10.1002/stem.2856 29781122 PMC6407639

[56] Boudreau LH Duchez AC Cloutier N Soulet D Martin N Bollinger J. Platelets release mitochondria serving as substrate for bactericidal group IIA-secreted phospholipase A2 to promote inflammation. Blood. (2014) 124:2173–83. 10.1182/blood-2014-05-573543 25082876 PMC4260364

[57] Pacak CA Preble JM Kondo H Seibel P Levitsky S Del Nido PJ. Actin-dependent mitochondrial internalization in cardiomyocytes: evidence for rescue of mitochondrial function. Biol Open. (2015) 4:622–6. 10.1242/bio.201511478 25862247 PMC4434813

[58] Kitani T Kami D Matoba S Gojo S. Internalization of isolated functional mitochondria: involvement of macropinocytosis. J Cell Mol Med. (2014) 18:1694–703. 10.1111/jcmm.12316 24912369 PMC4190914

[59] Kesner EE Saada-Reich A Lorberboum-Galski H. Characteristics of mitochondrial transformation into human cells. Sci Rep. (2016) 6:26057. 10.1038/srep26057 27184109 PMC4868981

[60] Alvarez-Dolado M Pardal R Garcia-Verdugo JM Fike JR Lee HO Pfeffer K. Fusion of bone-marrow-derived cells with Purkinje neurons, cardiomyocytes and hepatocytes. Nature. (2003) 425:968–73. 10.1038/nature02069 14555960

[61] Louwagie EJ Larsen TD Wachal AL Gandy TCT Baack ML. Mitochondrial transfer improves cardiomyocyte bioenergetics and viability in male rats exposed to pregestational diabetes. Int J Mol Sci. (2021) 22:2382. 10.3390/ijms22052382 33673574 PMC7956857

[62] Cowan DB Yao R Thedsanamoorthy JK Zurakowski D Del Nido PJ McCully JD. Transit and integration of extracellular mitochondria in human heart cells. Sci Rep. (2017) 7:17450. 10.1038/s41598-017-17813-0 29234096 PMC5727261

[63] Tian R Colucci WS Arany Z Bachschmid MM Ballinger SW Boudina S. Unlocking the secrets of mitochondria in the cardiovascular system: path to a cure in heart failure-a report from the 2018 National Heart, Lung, and Blood Institute Workshop. Circulation. (2019) 140:1205–16. 10.1161/CIRCULATIONAHA.119.040551 31769940 PMC6880654

[64] Zhou XL Wu X Xu QR Zhu RR Xu H Li YY. Notch1 provides myocardial protection by improving mitochondrial quality control. J Cell Physiol. (2019) 234:11835–41. 10.1002/jcp.27892 30515819

[65] Zhou H Wang S Zhu P Hu S Chen Y Ren J. Empagliflozin rescues diabetic myocardial microvascular injury via AMPK-mediated inhibition of mitochondrial fission. Redox Biol. (2018) 15:335–46. 10.1016/j.redox.2017.12.019 29306791 PMC5756062

[66] Chang JC Chang HS Wu YC Cheng WL Lin TT Chang HJ. Mitochondrial transplantation regulates antitumour activity, chemoresistance and mitochondrial dynamics in breast cancer. J Exp Clin Cancer Res. (2019) 38:30. 10.1186/s13046-019-1028-z 30674338 PMC6343292

[67] Lavorato M Iyer VR Dewight W Cupo RR Debattisti V Gomez L. Increased mitochondrial nanotunneling activity, induced by calcium imbalance, affects intermitochondrial matrix exchanges. Proc Natl Acad Sci USA. (2017) 114:E849–58. 10.1073/pnas.1617788113 28096415 PMC5293110

[68] Huang X Sun L Ji S Zhao T Zhang W Xu J. Kissing and nanotunneling mediate intermitochondrial communication in the heart. Proc Natl Acad Sci USA. (2013) 110:2846–51. 10.1073/pnas.1300741110 23386722 PMC3581932

[69] Maeda H Kami D Maeda R Murata Y Jo JI Kitani T. TAT-dextran-mediated mitochondrial transfer enhances recovery from models of reperfusion injury in cultured cardiomyocytes. J Cell Mol Med. (2020) 24:5007–20. 10.1111/jcmm.15120 32212298 PMC7205789

[70] Paliwal S Chaudhuri R Agrawal A Mohanty S. Human tissue-specific MSCs demonstrate differential mitochondria transfer abilities that may determine their regenerative abilities. Stem Cell Res Ther. (2018) 9:298. 10.1186/s13287-018-1012-0 30409230 PMC6225697

[71] McCully JD Levitsky S Del Nido PJ Cowan DB. Mitochondrial transplantation for therapeutic use. Clin Transl Med. (2016) 5:16. 10.1186/s40169-016-0095-4 27130633 PMC4851669

[72] Zhou P Pu WT. Recounting cardiac cellular composition. Circ Res. (2016) 118:368–70. 10.1161/CIRCRESAHA.116.308139 26846633 PMC4755297

[73] Masuzawa A Black KM Pacak CA Ericsson M Barnett RJ Drumm C. Transplantation of autologously derived mitochondria protects the heart from ischemia-reperfusion injury. Am J Physiol Heart Circ Physiol. (2013) 304:H966–82. 10.1152/ajpheart.00883.2012 23355340 PMC3625892

[74] Ali Pour P Kenney MC Kheradvar A. Bioenergetics consequences of mitochondrial transplantation in cardiomyocytes. J Am Heart Assoc. (2020) 9:e014501. 10.1161/JAHA.119.014501 32200731 PMC7428632

[75] Suen J Thomas J Kranz A Vun S Miller M. Effect of flavonoids on oxidative stress and inflammation in adults at risk of cardiovascular disease: a systematic review. Healthcare. (2016) 4:69. 10.3390/healthcare4030069 27649255 PMC5041070

[76] Al-Rawi NH Shahid AM. Oxidative stress, antioxidants, and lipid profile in the serum and saliva of individuals with coronary heart disease: is there a link with periodontal health? Minerva Stomatol. (2017) 66:212–25. 10.23736/S0026-4970.17.04062-6 28707864

[77] Doroszko A Dobrowolski P Radziwon-Balicka A Skomro R. New insights into the role of oxidative stress in onset of cardiovascular disease. Oxid Med Cell Longev. (2018) 2018:9563831. 10.1155/2018/9563831 29849927 PMC5924969

[78] Steven S Frenis K Oelze M Kalinovic S Kuntic M Bayo Jimenez MT. Vascular inflammation and oxidative stress: major triggers for cardiovascular disease. Oxid Med Cell Longev. (2019) 2019:7092151. 10.1155/2019/7092151 31341533 PMC6612399

[79] Karbach S Wenzel P Waisman A Munzel T Daiber A. eNOS uncoupling in cardiovascular diseases–the role of oxidative stress and inflammation. Curr Pharm Des. (2014) 20:3579–94. 10.2174/13816128113196660748 24180381

[80] Del Re DP Amgalan D Linkermann A Liu Q Kitsis RN. Fundamental mechanisms of regulated cell death and implications for heart disease. Physiol Rev. (2019) 99:1765–817. 10.1152/physrev.00022.2018 31364924 PMC6890986

[81] Heldman AW DiFede DL Fishman JE Zambrano JP Trachtenberg BH Karantalis V. Transendocardial mesenchymal stem cells and mononuclear bone marrow cells for ischemic cardiomyopathy: the TAC-HFT randomized trial. JAMA. (2014) 311:62–73. 10.1001/jama.2013.282909 24247587 PMC4111133

[82] Trachtenberg B Velazquez DL Williams AR McNiece I Fishman J Nguyen K. Rationale and design of the transendocardial injection of autologous human cells (bone marrow or mesenchymal) in chronic ischemic left ventricular dysfunction and heart failure secondary to myocardial infarction (TAC-HFT) trial: a randomized, double-blind, placebo-controlled study of safety and efficacy. Am Heart J. (2011) 161:487–93. 10.1016/j.ahj.2010.11.024 21392602

[83] Williams AR Trachtenberg B Velazquez DL McNiece I Altman P Rouy D. Intramyocardial stem cell injection in patients with ischemic cardiomyopathy: functional recovery and reverse remodeling. Circ Res. (2011) 108:792–6. 10.1161/CIRCRESAHA.111.242610 21415390 PMC3390160

[84] Malliaras K Li TS Luthringer D Terrovitis J Cheng K Chakravarty T. Safety and efficacy of allogeneic cell therapy in infarcted rats transplanted with mismatched cardiosphere-derived cells. Circulation. (2012) 125:100–12. 10.1161/CIRCULATIONAHA.111.042598 22086878 PMC3256094

[85] McCully JD Cowan DB Pacak CA Toumpoulis IK Dayalan H Levitsky S. Injection of isolated mitochondria during early reperfusion for cardioprotection. Am J Physiol Heart Circ Physiol. (2009) 296:H94–H105. 10.1152/ajpheart.00567.2008 18978192 PMC2637784

[86] Hsu CH Roan JN Fang SY Chiu MH Cheng TT Huang CC. Transplantation of viable mitochondria improves right ventricular performance and pulmonary artery remodeling in rats with pulmonary arterial hypertension. J Thorac Cardiovasc Surg. (2020) S0022-5223:32372-2. 10.1016/j.jtcvs.2020.08.014 32948302

[87] Kaza AK Wamala I Friehs I Kuebler JD Rathod RH Berra I. Myocardial rescue with autologous mitochondrial transplantation in a porcine model of ischemia/reperfusion. J Thorac Cardiovasc Surg. (2017) 153:934–43. 10.1016/j.jtcvs.2016.10.077 27938904

[88] Cowan DB Yao R Akurathi V Snay ER Thedsanamoorthy JK Zurakowski D. Intracoronary delivery of mitochondria to the ischemic heart for cardioprotection. Plos One. (2016) 11:e0160889. 10.1371/journal.pone.0160889 27500955 PMC4976938

[89] Blitzer D Guariento A Doulamis IP Shin B Moskowitzova K Barbieri GR. Delayed transplantation of autologous mitochondria for cardioprotection in a porcine model. Ann Thorac Surg. (2020) 109:711–9. 10.1016/j.athoracsur.2019.06.075 31421103

[90] Guariento A Blitzer D Doulamis I Shin B Moskowitzova K Orfany A. Preischemic autologous mitochondrial transplantation by intracoronary injection for myocardial protection. J Thorac Cardiovasc Surg. (2020) 160:e15–e29. 10.1016/j.jtcvs.2019.06.111 31564546

[91] Shin B Saeed MY Esch JJ Guariento A Blitzer D Moskowitzova K. A novel biological strategy for myocardial protection by intracoronary delivery of mitochondria: safety and efficacy. JACC Basic Transl Sci. (2019) 4:871–88. 10.1016/j.jacbts.2019.08.007 31909298 PMC6938990

[92] Guariento A Doulamis IP Duignan T Kido T Regan WL Saeed MY. Mitochondrial transplantation for myocardial protection in ex-situperfused hearts donated after circulatory death. J Heart Lung Transplant. (2020) S1053-2498:31625-9. 10.1016/j.healun.2020.01.1319 32703639

[93] Doulamis IP Guariento A Duignan T Orfany A Kido T Zurakowski D. Mitochondrial transplantation for myocardial protection in diabetic hearts. Eur J Cardiothorac Surg. (2020) 57:836–45. 10.1093/ejcts/ezz326 31782771

[94] Moskowitzova K Shin B Liu K Ramirez-Barbieri G Guariento A Blitzer D. Mitochondrial transplantation prolongs cold ischemia time in murine heart transplantation. J Heart Lung Transplant. (2019) 38:92–9. 10.1016/j.healun.2018.09.025 30391192 PMC6574228

[95] Weixler V Lapusca R Grangl G Guariento A Saeed MY Cowan DB. Autogenous mitochondria transplantation for treatment of right heart failure. J Thorac Cardiovasc Surg. (2021) 162:e111–21. 10.1016/j.jtcvs.2020.08.011 32919774

[96] Sid-Otmane C Perrault LP Ly HQ. Mesenchymal stem cell mediates cardiac repair through autocrine, paracrine and endocrine axes. J Transl Med. (2020) 18:336. 10.1186/s12967-020-02504-8 32873307 PMC7466793

[97] Bagno L Hatzistergos KE Balkan W Hare JM. Mesenchymal stem cell-based therapy for cardiovascular disease: progress and challenges. Mol Ther. (2018) 26:1610–23. 10.1016/j.ymthe.2018.05.009 29807782 PMC6037203

[98] Macia E Boyden PA. Stem cell therapy is proarrhythmic. Circulation. (2009) 119:1814–23. 10.1161/CIRCULATIONAHA.108.779900 19349334 PMC2739413

[99] Saei Arezoumand K Alizadeh E Pilehvar-Soltanahmadi Y Esmaeillou M Zarghami N. An overview on different strategies for the stemness maintenance of MSCs. Artif Cells Nanomed Biotechnol. (2017) 45:1255–71. 10.1080/21691401.2016.1246452 27809596

[100] Furlani D Ugurlucan M Ong L Bieback K Pittermann E Westien I. Is the intravascular administration of mesenchymal stem cells safe? Mesenchymal stem cells and intravital microscopy. Microvasc Res. (2009) 77:370–6. 10.1016/j.mvr.2009.02.001 19249320

[101] Emani SM Piekarski BL Harrild D Del Nido PJ McCully JD. Autologous mitochondrial transplantation for dysfunction after ischemia-reperfusion injury. J Thorac Cardiovasc Surg. (2017) 154:286–9. 10.1016/j.jtcvs.2017.02.018 28283239

[102] Fu A Shi X Zhang H Fu B. Mitotherapy for fatty liver by intravenous administration of exogenous mitochondria in male mice. Front Pharmacol. (2017) 8:241. 10.3389/fphar.2017.00241 28536524 PMC5422541

[103] Shi X Zhao M Fu C Fu A. Intravenous administration of mitochondria for treating experimental Parkinson's disease. Mitochondrion. (2017) 34:91–100. 10.1016/j.mito.2017.02.005 28242362

[104] Mobarrez F Fuzzi E Gunnarsson I Larsson A Eketjall S Pisetsky DS. Microparticles in the blood of patients with SLE: size, content of mitochondria and role in circulating immune complexes. J Autoimmun. (2019) 102:142–9. 10.1016/j.jaut.2019.05.003 31103269

[105] Herrmann IK Wood MJA Fuhrmann G. Extracellular vesicles as a next-generation drug delivery platform. Nat Nanotechnol. (2021) 16:748–59. 10.1038/s41565-021-00931-2 34211166

[106] Cai J Wu J Wang J Li Y Hu X Luo S. Extracellular vesicles derived from different sources of mesenchymal stem cells: therapeutic effects and translational potential. Cell Biosci. (2020) 10:69. 10.1186/s13578-020-00427-x 32483483 PMC7245623

[107] Sherman CD Lodha S Sahoo S. EV cargo sorting in therapeutic development for cardiovascular disease. Cells. (2021) 10:1500. 10.3390/cells10061500 34203713 PMC8232200

[108] Bolli R Tang XL Sanganalmath SK Rimoldi O Mosna F Abdel-Latif A. Intracoronary delivery of autologous cardiac stem cells improves cardiac function in a porcine model of chronic ischemic cardiomyopathy. Circulation. (2013) 128:122–31. 10.1161/CIRCULATIONAHA.112.001075 23757309 PMC3807652

[109] Adamiak M Cheng G Bobis-Wozowicz S Zhao L Kedracka-Krok S Samanta A. Induced pluripotent stem cell (iPSC)-derived extracellular vesicles are safer and more effective for cardiac repair than iPSCs. Circ Res. (2018) 122:296–309. 10.1161/CIRCRESAHA.117.311769 29118058 PMC5775034

[110] Wang X Chen Y Zhao Z Meng Q Yu Y Sun J. Engineered exosomes with ischemic myocardium-targeting peptide for targeted therapy in myocardial infarction. J Am Heart Assoc. (2018) 7:e008737. 10.1161/JAHA.118.008737 30371236 PMC6201471

[111] Falk MJ Decherney A Kahn JP. Mitochondrial replacement techniques–implications for the clinical community. N Engl J Med. (2016) 374:1103–6. 10.1056/NEJMp1600893 26910290 PMC4936492

[112] Dorji J Vander Jagt CJ Garner JB Marett LC Mason BA Reich CM. Expression of mitochondrial protein genes encoded by nuclear and mitochondrial genomes correlate with energy metabolism in dairy cattle. BMC Genomics. (2020) 21:720. 10.1186/s12864-020-07018-7 33076826 PMC7574280

[113] Meiklejohn CD Holmbeck MA Siddiq MA Abt DN Rand DM Montooth KL. An incompatibility between a mitochondrial tRNA and its nuclear-encoded tRNA synthetase compromises development and fitness in Drosophila. PLoS Genet. (2013) 9:e1003238. 10.1371/journal.pgen.1003238 23382693 PMC3561102

[114] Trier CN Hermansen JS Saetre GP Bailey RI. Evidence for mito-nuclear and sex-linked reproductive barriers between the hybrid Italian sparrow and its parent species. PLoS Genet. (2014) 10:e1004075. 10.1371/journal.pgen.1004075 24415954 PMC3886922

[115] Ma H Marti Gutierrez N Morey R Van Dyken C Kang E Hayama T. Incompatibility between nuclear and mitochondrial genomes contributes to an interspecies reproductive barrier. Cell Metab. (2016) 24:283–94. 10.1016/j.cmet.2016.06.012 27425585 PMC4981548

[116] Caicedo A Aponte PM Cabrera F Hidalgo C Khoury M. Artificial mitochondria transfer: current challenges, advances, and future applications. Stem Cells Int. (2017) 2017:7610414. 10.1155/2017/7610414 28751917 PMC5511681

[117] Mitalipov S Wolf DP. Clinical and ethical implications of mitochondrial gene transfer. Trends Endocrinol Metab. (2014) 25:5–7. 10.1016/j.tem.2013.09.001 24373414 PMC4005369

[118] Xu S Schaack S Seyfert A Choi E Lynch M Cristescu ME. High mutation rates in the mitochondrial genomes of Daphnia pulex. Mol Biol Evol. (2012) 29:763–9. 10.1093/molbev/msr243 21998274 PMC3350313

[119] Picard M Taivassalo T Ritchie D Wright KJ Thomas MM Romestaing C. Mitochondrial structure and function are disrupted by standard isolation methods. PLoS ONE. (2011) 6:e18317. 10.1371/journal.pone.0018317 21512578 PMC3065478

[120] Busch KB Kowald A Spelbrink JN. Quality matters: how does mitochondrial network dynamics and quality control impact on mtDNA integrity? Philos Trans R Soc Lond B Biol Sci. (2014) 369:20130442. 10.1098/rstb.2013.0442 24864312 PMC4032518

[121] Mustafa MF Fakurazi S Abdullah MA Maniam S. Pathogenic mitochondria DNA mutations: current detection tools and interventions. Genes. (2020) 11:192. 10.3390/genes11020192 32059522 PMC7074468

[122] Aryaman J Johnston IG Jones NS. Mitochondrial heterogeneity. Front Genet. (2018) 9:718. 10.3389/fgene.2018.00718 30740126 PMC6355694

[123] Wu S Zhang A Li S Chatterjee S Qi R Segura-Ibarra V. Polymer functionalization of isolated mitochondria for cellular transplantation and metabolic phenotype alteration. Adv Sci. (2018) 5:1700530. 10.1002/advs.201700530 29593955 PMC5867055

[124] Newson AJ Wilkinson S Wrigley A. Ethical and legal issues in mitochondrial transfer. EMBO Mol Med. (2016) 8:589–91. 10.15252/emmm.201606281 27137493 PMC4888849

